# The Role of Nerve Growth Factor on the Ocular Surface: A Review of the Current Experimental Research and Clinical Practices

**DOI:** 10.3390/ijms26136012

**Published:** 2025-06-23

**Authors:** Nicolás Kahuam-López, Amir Hosseini, Jennifer Y. M. Ling, Joseph Chiang, Alfonso Iovieno, Sonia N. Yeung

**Affiliations:** Department of Ophthalmology and Visual Sciences, University of British Columbia, Vancouver, BC V5Z 3N9, Canada

**Keywords:** ocular surface, nerve growth factor, cenegermin, corneal wound healing, allergic conjunctivitis, neurotrophic keratopathy, dry eye disease, corneal transplant, refractive surgery, cataract surgery

## Abstract

The ocular surface is susceptible to a wide spectrum of inflammatory, degenerative, and neurotrophic diseases that can impair vision. The complex pathophysiology and limited therapeutic options associated with these conditions continue to pose significant clinical challenges. Nerve Growth Factor (NGF), a neurotrophin initially recognized for its role in neuronal survival and differentiation, has emerged as a key regulator of ocular surface homeostasis and repair. Beyond its neurotrophic functions, NGF is suggested to influence epithelial proliferation, immune responses, tear secretion, and angiogenesis. Experimental and clinical studies have implicated NGF in both the pathogenesis and potential treatment of various ocular surface diseases, including allergic conjunctivitis, neurotrophic keratopathy (NK), immune-mediated and herpetic keratitis, and dry eye disease (DED), as well as post-surgical corneal wound healing. Notably, recombinant human NGF (rhNGF, cenegermin) has been approved as the first topical biologic therapy for NK. Despite encouraging clinical outcomes, challenges such as high treatment costs, limited long-term data, and potential proangiogenic effects remain. This review consolidates current evidence on the role of NGF in ocular surface health and disease, highlighting its biological mechanisms, clinical applications, and future therapeutic potential.

## 1. Introduction

Nerve Growth Factor (NGF) is a homodimeric protein initially discovered in the peripheral nervous system and named for its effects on neuron growth and differentiation [1]. Since its discovery, it has been shown to play a role in numerous physiological processes and pathological conditions. On the ocular surface, NGF has been studied in the context of allergic conjunctivitis [2,3,4,5], NK [6,7,8,9,10,11,12], immune and infectious keratitis [13,14,15,16], DED [17,18,19], corneal transplant [20,21], cataract [22], and refractive surgery [23,24,25]. This review discusses the current experimental research and clinical practices regarding NGF on the ocular surface.

In the sections that follow, we begin by examining the molecular biology of NGF, including its gene structure, proteolytic processing, and downstream signaling via tyrosine protein kinase receptor-A (TrkA^NGFR^) and p75 pan-NT receptor, named p75^NTR^. These receptors activate pathways that regulate diverse cellular processes such as neuronal survival, apoptosis, calcium homeostasis, inflammation, immune cell recruitment, cell proliferation, and tissue remodeling [26,27,28,29,30,31,32,33,34,35,36,37,38]. The ocular expression of NGF receptors is then reviewed, with emphasis on their localization in both neuronal and non-neuronal cells [38,39,40,41,42,43,44,45,46,47,48], supporting NGF’s diverse effects.

Building on this foundation, we examine the physiological roles of NGF at the ocular surface, focusing on its contribution to epithelial cell proliferation and differentiation, corneal nerve regeneration, and modulation of local immune responses. To support this, we present ocular-surface-specific in vitro data, demonstrating the effects of exogenous NGF treatment on goblet cell expression and secretion, mucin production and release, conjunctival epithelial cell differentiation [45,49], and corneal epithelial migration and proliferation [36]. We also highlight the upregulation of the TrkA^NGFR^ receptor and matrix metalloproteinase-9 (MMP-9) expression [36,37]. Furthermore, we explore the induction of fibroblastic-keratocyte and conjunctival fibroblast differentiation into myofibroblasts and enhanced migration upon exogenous NGF treatment [48,50].

We then synthesize evidence from animal models and clinical studies evaluating NGF’s therapeutic applications in a range of ocular surface diseases. Particular emphasis is placed on NK, a rare but vision-threatening disorder affecting approximately 5 individuals or fewer in 10,000 [51], for which topical rhNGF (cenegermin) remains the only FDA-approved pharmacologic therapy [52]. We also briefly compare NGF therapy with other conventional NK treatment options, such as tear substitutes, amniotic membrane transplantation, neurotization, and autologous serum drops, highlighting why NGF therapy offers great potential as a therapeutic approach to target the underlying neurotrophic deficit.

Lastly, we critically examine the limitations and unresolved questions surrounding NGF-based therapies, including the high cost and limited accessibility of rhNGF, its complex administration schedule, the scarcity of long-term safety and efficacy data, and mechanistic concerns such as the potential proangiogenic effects of NGF in corneal neovascularization [11,53]. We highlight specific knowledge gaps, such as the differential effects of NGF across various cell types and disease contexts, and emphasize the need for further multicenter randomized trials and mechanistic investigations to optimize NGF’s clinical utility and expand its approved therapeutic indications.

## 2. Methods of Literature Search

A comprehensive literature search was performed using PubMed and Embase to identify studies examining the role of NGF in ocular surface diseases.

The PubMed search used the following terms: (“Nerve Growth Factor” or “NGF” or “Cenegermin”) combined with (“Cornea” or “Ocular Surface” or “Pterygium Of Conjunctiva And Cornea” or “Pterygium” or “Conjunctiva” or “Dry Eye Syndrome” or “Dry Eye” or “Dry Eye Disease” or “Conjunctivitis Sicca” or “Keratoconjunctivitis Sicca” or “Keratitis Sicca” or “Keratitis” or “Immune Keratitis” or “Herpetic Keratitis” or “Corneal Ulcer” or “Refractive Surgical Procedures” or “Refractive Surgery” or “LASIK” or “Laser-assisted In Situ Keratomileusis” or “PRK” or “Photorefractive Keratectomy” or “SMILE” or “Small Incision Lenticule Extraction” or “Corneal Surgery” or “Corneal Transplantation” or “Corneal Transplant” or “Corneal Neovascularization” or “Corneal Diseases” or “Graft Rejection” or “Graves Ophthalmopathy” or “Thyroid Eye Disease” or “Allergic Conjunctivitis” or “Vernal Keratoconjunctivitis” or “Conjunctivitis” or “Neuralgia” or “Neuropathic Pain”).

For Embase, a two-step search was conducted. First, ocular surface and corneal disease terms were searched using the following: (“Cornea” or “Ocular Surface” or “Conjunctiva” or “Pterygium Of Conjunctiva” or “Pterygium Of Cornea” or “Pterygium” or “Dry Eye Syndrome” or “Dry Eye” or “Dry Eye Disease” or “Conjunctivitis Sicca” or “Keratoconjunctivitis Sicca” or “Keratitis Sicca” or “Keratitis” or “Immune Keratitis” or “Herpetic Keratitis” or “Corneal Ulcer” or “Refractive Surgical Procedures” or “Refractive Surgery” or “LASIK” or “Laser-assisted In Situ Keratomileusis” or “PRK” or “Photorefractive Keratectomy” or “SMILE” or “Small Incision Lenticule Extraction” or “Corneal Surgery” or “Corneal Transplantation” or “Corneal Transplant” or “Graft Rejection” or “Corneal Neovascularization” or “Corneal Diseases” or “Graves Ophthalmopathy” or “Thyroid Eye Disease” or “Allergic Conjunctivitis” or “Vernal Keratoconjunctivitis” or “Conjunctivitis” or “Neuralgia” or “Neuropathic Pain”). These were then combined with NGF-related terms: (“Nerve Growth Factor” or “NGF” or “Cenegermin”). The final search strategy was constructed using the Boolean operator AND to retrieve records containing both sets of terms.

## 3. Biology of Nerve Growth Factor

NGF is a key neurotrophic factor essential for the development and maintenance of both the central and peripheral nervous systems [54]. It plays a critical role in neuronal repair and apoptosis, which are involved in various neurological disorders [55,56]. NGF is also thought to contribute to several other biological processes, including immune regulation, inflammation, pulmonary hypertension, wound healing, and cancer metastasis [54,56,57,58,59]. Furthermore, NGF has several therapeutic applications in neurological, ocular, and skin diseases [13,60,61,62,63,64,65,66,67,68,69,70,71,72,73,74].

**Figure 1:**
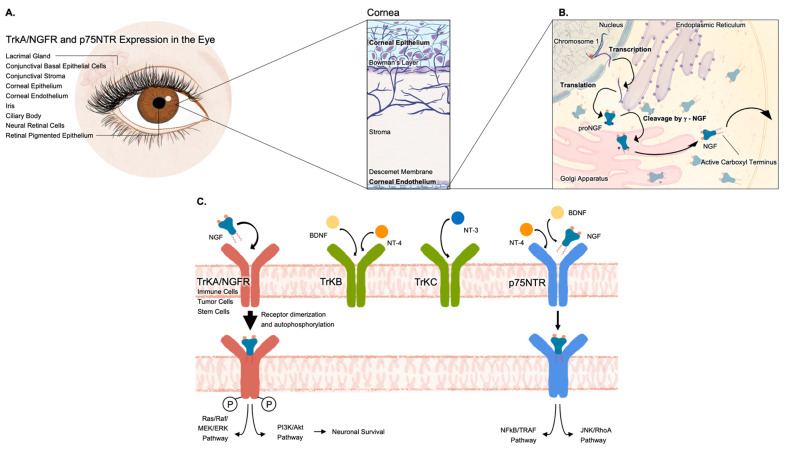
Schematic overview of nerve growth factor in the eye. (**A**) TrkA/NGFR and p75^NTR^ are expressed in the lacrimal gland, conjunctival basal epithelial cells, conjunctival stroma, corneal epithelium, corneal endothelium, iris, ciliary body, neural retinal cells, and retinal pigmented epithelium. (**B**) NGF synthesis and cleavage: the endoplasmic reticulum synthesizes proNGF, which is then folded and transferred to the Golgi apparatus. Intracellular cleavage of proNGF occurs by the calcium-dependent serine protease of its γ-subunits, exposing the biologically active carboxyl terminus. Additional proNGF is released from the cells, and active NGF is formed through the action of extracellular proteases. (**C**) TrkA/NGFR and p75^NTR^ signal transduction pathways: upon NGF binding to TrkA/NGFR, TrkA/NGFR dimerizes and is autophosphorylated at its intracellular domain, resulting in the activation of the Ras–Raf–MEK–ERK and PI3K–AKT signaling pathways. When NGF binds to the receptor p75^NTR^, it activates the JNK or NFκB signaling pathways, including downstream interactions with RhoA and TRAF proteins.

NGF was discovered in 1950 by Rita Levi-Montalcini. It was characterized by its ability to stimulate growth, survival, and differentiation of neurons during development and after injury [38,74]. Since its discovery, NGF has been found to be produced by epithelial cells, fibroblasts/myofibroblasts, endothelial cells, smooth muscle cells, hepatocytes, glial cells, astrocytes, Müller cells, antigen-presenting cells, lymphocytes, granulocytes, mast cells, and eosinophils [75]. The NGF gene is located on the short arm of chromosome 1 at 1p13.2 [74,76,77,78]. It is initially produced as a 118-amino-acid glycoprotein, formed by the subunits α2, β, and γ2, bound by non-covalent bonds. The endoplasmic reticulum synthesizes NGF in its prosomal form (proNGF), which is then folded and transferred to the Golgi apparatus (Figure 1). The majority of active NGF results from the intracellular cleavage of proNGF by the calcium-dependent serine protease of its γ-subunits, exposing the biologically active carboxyl terminus. Some additional proNGF is released by the cells and exists as active NGF after processing by extracellular proteases [54,59].

NGF is part of the neurotrophin family that includes brain-derived neurotrophic factor (BDNF), neurotrophin-3 (NT-3), NT-4/5, and NT-6 [79]. These neurotrophins exert their biological functions through three Trk receptors: NGF binds to the tropomyosin TrkA^NGFR^ tyrosine kinase receptor (termed p140TrkA^NGFR^ or TrkA^NGFR^), BDNF and NT-4 bind to TrkB, and NT-3 binds to TrkC. All neurotrophins also bind with lower affinity to the p75^NTR^ receptor [38,80].

NGF’s neurotrophic effects are primarily mediated by the TrkA^NGFR^ receptor, and its secondary effects are mediated by the p75^NTR^ receptor [81,82]. TrkA^NGFR^ consists of a 140 kD molecule that forms a receptor with three domains: an extracellular receptor domain, a single transmembrane helix, and an intracellular tyrosine kinase domain [83]. The extracellular sequence is composed of five additional domains (D1–D5). D5 is an immunoglobulin-like domain responsible for NGF binding [84,85]. TrkA^NGFR^ is extensively expressed in neuronal and non-neuronal tissues, such as immune cells, tumor cells, and stem cells [86,87].

When NGF binds to TrkA^NGFR^, TrkA^NGFR^ dimerizes and is autophosphorylated at its intracellular domain, resulting in the activation of signaling pathways rat sarcoma protein (Ras), serine/threonine kinase (Raf), mitogen-activated protein kinase (MEK), extracellular-signal-regulated kinase (ERK), phosphoinositide 3-kinases (PI3K), and protein kinase B (AKT), imperative to neuronal survival [26,79,88,89]. When added exogenously to cultured cells, NGF binding can trigger pro-survival downstream signaling cascades that are critical for regulating neuronal growth, differentiation, and survival [90,91,92,93,94]. Specifically, PI3K/AKT signaling and MEK/ERK signaling suppress apoptosis by inhibiting caspase-3 activation and promote axonal regeneration and synaptic plasticity [90,94,95]. In cultured neurons, NGF also reduces oxidative stress, enhances antioxidant activity, and modulates microglial polarization toward an anti-inflammatory phenotype [90,96].

On the other hand, the union of NGF and p75^NTR^ receptor activates the c-Jun N-terminal kinase (JNK) or nuclear factor kappa B (NFκB) signaling pathways, including downstream interactions with Ras homolog family member A (RhoA) and tumor-necrosis-factor-receptor-associated factor (TRAF) proteins [26,79,88,89]. Binding of NGF to p75^NTR^, especially in the absence of TrkA^NGFR^, can trigger apoptotic signaling through the JNK and NF-κB pathways, thereby underscoring the critical role of cellular context and receptor availability in determining the biological outcomes of NGF signaling [75,90,97].

Both TrkA^NGFR^ and p75^NTR^ are expressed in the basal epithelial cells, goblet cells, fibroblasts, and the stroma of the conjunctiva; limbal epithelial cells; corneal keratocytes and epithelial and endothelial cells; the iris; ciliary body; retinal pigmented epithelium; cells of the neural retina; and the lacrimal gland [39,40,41,42,43,44,45,46,47,48,98,99]. Given this, TrkA^NGFR^ and p75^NTR^ expression extends beyond neuronal elements to include resident epithelial, endothelial, stromal, and secretory cells, underscoring NGF’s broader role in non-neuronal ocular functions. Moreover, TrkA^NGFR^ and p75^NTR^ have also been detected in skin and lung fibroblasts in vitro [100], providing additional evidence of their non-neuronal expression and activity.

## 4. The Role of Nerve Growth Factor on the Ocular Surface

The role of TrkA^NGFR^ in ocular surface diseases was first proposed by Lambiase et al. (1998) [39], who examined receptor expression under both normal and pathological conditions. The authors reported elevated levels of TrkA^NGFR^ in patients with vernal keratoconjunctivitis (VKC) and ocular cicatricial pemphigoid (OCP) compared to healthy individuals. In a later study, Micera et al. (2015) [99] further characterized NGF signaling in OCP. OCP conjunctivas showed αSMA-expressing fibroblasts and high NGF levels. Advanced-stage fibroblasts exhibited increased p75^NTR^ and reduced TrkA^NGFR^ expression compared to early-stage cells. NGF exposure reduced αSMA, p75^NTR^, and cytokine release (TGF-β1, IL-4) in early, but not in advanced, OCP fibroblasts in vitro, suggesting stage-specific differences in NGF responsiveness. Increased receptor expression was also observed in corneal ulcers, suggesting a potential role for TrkA^NGFR^ in corneal epithelial remodeling. Furthermore, TrkA^NGFR^ is expressed in limbal basal epithelial cells in vivo and has been implicated in regulating the differentiation and proliferation of limbal stem cells [43,101]. Together, these findings support a role for NGF in promoting the proliferation and differentiation of limbal epithelial progenitor cells.

Under physiological conditions, NGF is released into the tear film and aqueous humor [41,67] and is suggested to originate from the corneal epithelium, stroma, and endothelial cells, as reported in studies of cadaveric human corneas [69]. Lambiase et al. (2002) [41] demonstrated the presence of NGF and TrkA^NGFR^ in the cornea, iris, ciliary body, and lens of rabbits, with the highest NGF levels found in the iris, suggesting that these molecules may play a role in regenerating sensory and sympathetic nerve fibers [102]. Furthermore, in the cornea, NGF accelerates keratocyte migration, stimulates immunomodulation and healing after injury, induces epithelial cell proliferation and differentiation, and maintains corneal epithelial stem cells [40,103]. Corneal innervation has also been proposed to be NGF-dependent by De Castro et al. (1998) [104], who demonstrated a relationship between NGF and corneal nerve density by analyzing the innervation pattern and response to noxious stimulation in TrkA^NGFR^ knockout mice. They observed reduced corneal nerve density and impaired corneal sensitivity in the knockout group compared to wild-type and heterozygous mice, further supporting the role of NGF in corneal innervation.

Furthermore, in vitro studies demonstrate that NGF exerts its biological effects on corneal epithelial cells, and neurons primarily through high-affinity binding to the TrkA^NGFR^ receptor. This activates key intracellular signaling cascades, particularly the PI3K/AKT and MEK/ERK pathways, which regulate cell survival, proliferation, and differentiation [94,105,106]. In human corneal epithelial cells, NGF stimulation leads to time- and dose-dependent phosphorylation of AKT and ERK, promoting cell cycle progression via upregulation of D-type cyclins and resulting in accelerated growth of these cells [105]. This aligns with broader evidence highlighting the role of PI3K/AKT signaling in supporting corneal epithelial homeostasis and wound healing [107].

In vivo, NGF is endogenously expressed in the murine cornea, with marked upregulation during reinnervation following surgical transection of corneal nerves [108]. Beyond its direct neurotrophic effects, NGF is also suggested to facilitate corneal healing by stimulating the release of pro-neural peptides such as substance P [68,109,110]. Supporting evidence from murine models also reveal that both NGF and glial-cell-line-derived neurotrophic factor (GDNF) are significantly upregulated in regenerating corneal epithelium, correlating with the reappearance of subbasal nerve fibers [111]. Conditioned medium from corneal epithelial cells enriched with NGF and GDNF promotes neurite outgrowth from trigeminal ganglion neurons, characterized by increased elongation and branching. Notably, neutralization of either NGF or GDNF significantly impairs this neuritogenic effect and diminishes corneal nerve regeneration in vivo. Furthermore, diabetic mice exhibit attenuated NGF and GDNF expression in regenerating corneal tissue, which is associated with delayed epithelial and nerve repair. This impairment can be rescued by exogenous supplementation of either growth factor [111]. Finally, functional studies using rats demonstrate that inhibition of endogenous NGF activity through anti-NGF antibodies delays corneal epithelial wound healing, whereas topical administration of exogenous NGF significantly accelerates epithelial closure [40].

NGF has also been suggested to play a role in modulating tear secretion and protecting against the deleterious effects of DED [47,49]. In primary human conjunctival epithelial cells and cell line cultures, NGF induces a dose-dependent increase in goblet cell numbers, promotes the production and storage of mucin-5AC precursor (MUC5AC), and promotes neural innervation and maintenance [49].

Additionally, NGF has been proposed to regulate immune cells infiltrating the cornea and conjunctiva during chronic inflammatory states associated with allergic and autoimmune diseases [39]. Lambiase et al. (1998) [39] demonstrated increased TrkA^NGFR^ expression on eosinophils and T-helper lymphocytes in conjunctival biopsies from patients with VKC. The detection of NGF receptors on immune cells at the ocular surface during inflammatory conditions further supports the proposed role of NGF in modulating inflammatory processes. Finally, NGF may also act as an angiogenic factor under pathological conditions. Ribatti et al. (2009) [112] demonstrated a correlation between microvascular density and NGF/TrkA^NGFR^ expression in endothelial cells of human pterygium. The study proposed that NGF is present and activates TrkA^NGFR^ in pterygium, suggesting its involvement in the angiogenic response characteristic of this condition.

## 5. Clinical Applications of Nerve Growth Factor on the Ocular Surface

NGF has been extensively studied in both experimental and clinical contexts for its role in ocular surface diseases, including allergic conjunctivitis, NK, immune and infectious keratitis, DED, and corneal transplantation, cataract, and refractive surgeries. This section summarizes the current understanding of NGF as a therapeutic target in these conditions (Table 1).

### 5.1. Allergic Conjunctivitis

Preliminary experimental evidence suggests that NGF plays an essential role in allergic reactions through its release by neurons, lymphocytes, mast cells, fibroblasts, and smooth muscle cells following antigenic stimulation [113,114]. During allergic conjunctivitis, NGF is released by conjunctival epithelial and immune cells. Upon binding to TrkA^NGFR^ and p75^NTR^, NGF modulates allergic responses via the p75^NTR^/JNK signaling pathway in conjunctival epithelial cells [115]. It is also hypothesized to actively contribute to allergic inflammation by binding to its receptors on eosinophils, mast cells, and T-helper lymphocytes [2,3,4].

Lambiase et al. (1995, 1997) [3,5] evaluated NGF plasma concentrations in patients with VKC and examined their correlation with plasma levels of substance P, as well as serum levels of eosinophil cationic protein (ECP) and immunoglobulin E. NGF plasma levels were measured, and tarsal and bulbar conjunctival biopsy specimens were collected from both VKC patients and healthy matched controls. The results showed significantly higher plasma concentrations of NGF in VKC patients, which correlated with the number of mast cells in the tarsal and bulbar conjunctiva.

The conjunctival epithelium and NGF may play an active role in ocular allergic inflammation through the p75^NTR^ pathway. Sacchetti et al. (2019) [115] investigated the involvement of TrkA^NGFR^ and p75^NTR^ in patients with allergic rhinoconjunctivitis (ARC) before and after a conjunctival provocation test (CPT) with an allergen. In the quiescent phase, ARC patients exhibited a significant increase in conjunctival p75^NTR^ expression, while NGF and TrkA^NGFR^ were undetectable. Following allergen challenge, patients showed a significant increase in tear NGF levels and upregulation of downstream p75^NTR^ signaling molecules, including JNK/stress-activated protein kinase (SAPK) and p53. These findings suggest an active role for p75^NTR^ in mediating allergic responses on the ocular surface.

### 5.2. Neurotrophic Keratopathy

Damage to corneal nerves alters the metabolism and survival of the epithelium by depleting the tissue of acetylcholine and substance P, consequently resulting in decreased epithelial mitosis and impaired epithelial healing, which may lead to the development of neurotrophic ulcers [116,117].

Murine NGF purified from submaxillary glands has been used to treat ocular surface diseases such as NK due to herpetic infections, chemical burns, topical anesthetic abuse, and diabetes [66,68]. In 1969, Bocchini and Angeletti [118] described a method for purifying NGF from adult mouse submaxillary glands. Prospective pilot studies later evaluated the effects of intracerebroventricular administration of murine growth factors in patients with Parkinson’s and Alzheimer’s disease [65,119]. Eriksdotter et al. (1998) [65] and Olson et al. (1991) [119] investigated the administration of murine NGF into the lateral cerebral ventricle and left putamen. The rationale behind these studies was to explore potential therapies that could counteract cholinergic degeneration and/or induce the formation of new cholinergic nerve terminals in Alzheimer’s disease, and to support chromaffin cell autografts in patients with severe Parkinson’s disease. Although these studies were insufficient to demonstrate the therapeutic intracerebral use of NGF in neurodegenerative diseases, they laid the foundation for future research into NGF’s clinical applications.

In 1998, Lambiase et al. [68] evaluated the effect of topical murine NGF in patients with neurotrophic ulcers and observed complete corneal healing in all patients after 10 days to 6 weeks of treatment. Additionally, an observational study by Bonini et al. (2000) [66] evaluated 45 eyes from 43 patients with stage 2 or 3 NK that were unresponsive to artificial tears and therapeutic contact lenses. Patients received murine NGF eye drops at a concentration of 200 µg/mL every two hours (from 6:00 AM to 12:00 AM) for two days, followed by one drop every four hours until complete epithelial healing. Once healed, a lower concentration (100 µg/mL) was administered four times daily for two weeks. Complete corneal healing was achieved in all patients within 2 to 6 weeks of initiating murine NGF treatment. Mild and transient ocular adverse effects (AEs), such as photophobia, burning sensation, and conjunctival hyperemia, were reported, but no systemic AEs occurred during the follow-up period of 16 to 72 months [13,66,120].

Topical rhNGF has also been studied for the treatment of NK. It is the only FDA-approved pharmacologic treatment for NK and the first topical biologic approved in the field of ophthalmology. rhNGF (cenegermin) ophthalmic solution 0.002% received FDA approval in 2018 and is synthesized using recombinant *Escherichia coli* [52].

A case of central NK in Wallenberg syndrome (WS), unresponsive to artificial tears and bandage contact lenses, was treated with rhNGF and reported by Mandarà et al. (2022) [7]. A 47-year-old man with WS, caused by a stroke in the territory of the left vertebrobasilar artery, developed left corneal NK grade 3 of central origin. The patient was treated with topical rhNGF at a concentration of 20 μg/mL, one drop six times daily for eight weeks. The corneal epithelium healed completely, with no recurrence at one-year follow-up. Another case report [8] described an 84-year-old female who developed NK in the context of diabetes mellitus, infectious keratitis, DED, and corneal exposure. She was treated with rhNGF 20 μg/mL, administered six times daily. The epithelial defect began to improve five weeks after initiating treatment and resolved completely after eight weeks.

The long-term clinical efficacy of topical rhNGF, formulated at 20 μg/mL, for the treatment of NK was demonstrated in a retrospective case series from a single center [9]. Eighteen patients with stage 2 or 3 NK were treated with rhNGF six times daily for eight weeks and followed for up to 48 months. All patients achieved complete corneal healing by the end of the treatment period. Three patients experienced recurrence of persistent epithelial defects within 12 months, and one patient experienced recurrence of a corneal ulcer within 36 months. The authors concluded that rhNGF was effective in restoring long-term corneal epithelial stability and highlighted the need for larger, long-term, prospective, controlled trials.

Two randomized controlled trials evaluated the safety and efficacy of rhNGF in patients with NK. Patients were administered 10 or 20 μg/mL rhNGF eye drops six times daily for eight weeks. The rhNGF-treated groups demonstrated both statistically and clinically significant reductions in lesion size and disease progression during treatment [10,11,12]. The REPARO trial [11,12], a multicenter randomized controlled trial, assessed the use of 10 and 20 μg/mL rhNGF eye drops in patients with stage 2 or 3 NK. The trial confirmed the safety and efficacy of rhNGF in moderate to severe cases. Phase I evaluated safety in 18 patients, while Phase II assessed safety and efficacy in 156 patients. Participants were randomized into three groups: 10 μg/mL rhNGF, 20 μg/mL rhNGF, or vehicle, for an 8-week treatment period. All participants received one eye drop six times daily. At week 8, 43.1% of vehicle-treated patients exhibited less than 0.5 mm of lesion staining compared to 74.5% in the 10 μg/mL rhNGF group and 74.0% in the 20 μg/mL rhNGF group. Another multicenter randomized, vehicle-controlled trial [10] evaluated 20 μg/mL rhNGF in NK patients, using complete healing of persistent epithelial defects or corneal ulcers at 8 weeks as the primary endpoint. Patients received one eye drop six times daily. The study found that 65.2% of patients treated with rhNGF achieved 0 mm of lesion staining with no residual staining compared to only 16.7% of vehicle-treated patients. Additionally, Cheung et al. (2022) [121] conducted a retrospective chart review demonstrating the effectiveness of rhNGF in combination with bandage contact lenses for NK. The study reported a 70% improvement in corneal sensation and complete epithelial healing in 67% of patients with persistent epithelial defects.

NK is infrequent in the pediatric population. Its etiology can be congenital, either as isolated corneal anesthesia or in association with systemic disease, or acquired, resulting from infections, neurological disorders, or the toxicity of topical medications [122]. Several case reports [123,124,125,126,127] and a retrospective case series [53] examined the use of rhNGF in the pediatric population. Papadopoulos et al. (2021) [123] reported the case of a 7-year-old male who developed NK following two years of treatment with antibiotics, steroids, and artificial tears for infectious keratitis. The patient was successfully treated with 20 μg/mL rhNGF eye drops administered six times daily for eight weeks, with no reported AEs. In another case [124], a 3-year-old male developed NK after undergoing surgery for rhabdomyosarcoma of the jaw. Treatment with 20 μg/mL rhNGF eye drops, administered six times daily for eight weeks, resulted in complete corneal epithelial healing within three weeks. A separate case report [127] described a 5-month-old child with congenital corneal anesthesia who developed NK and was treated with 200 μg/mL murine NGF, administered every two hours (from 6:00 AM to 12:00 AM) for two days. This was followed by one drop six times daily until epithelial healing. Once the corneal epithelium was intact, the child received 100 μg/mL murine NGF, administered six times daily for six months. By day 10 of treatment, the mother reported that the child began displaying signs of discomfort upon instillation of the drops, which was postulated to indicate the return of corneal sensation. In a different study, Fausto et al. (2020) [125] reported the case of a 9-year-old patient with pontine tegmental cap dysplasia and bilateral cranial nerve VI and VIII palsies who developed NK and was treated with 20 μg/mL rhNGF, one drop six times daily at two-hour intervals (from 8 AM to 8 PM). After eight weeks of treatment, the authors reported complete epithelial healing and a reduction in corneal opacity and neovascularization. Cochet–Bonnet corneal esthesiometry could not be performed due to the patient’s lack of cooperation. No local or systemic AEs were observed. Finally, a retrospective case series [53] involving eight pediatric patients (age range 2 to 18 years) from three tertiary referral institutions treated NK with 20 µg/mL rhNGF. The etiologies of NK included Stickler syndrome (cranial nerve V palsy), surgery for cerebellopontine tumor, cranial nerve V agenesis, trauma (fireworks injury), complex regional pain syndrome, Gómez–López-Hernández syndrome, Stevens–Johnson syndrome, and Herpes Simplex Virus (HSV) keratitis. The authors reported improvement in corneal sensation (Cochet–Bonnet esthesiometry) and Mackie classification staging in 63% of patients. All patients in this series had previously received NK-specific therapies (e.g., tarsorrhaphy, amniotic membrane transplant, autologous serum eye drops). AEs reported during therapy included ocular pain, difficulty sleeping, and continued corneal thinning.

To conclude, rhNGF is unique among current therapies for NK in that it directly targets the underlying pathophysiological deficit: corneal nerve damage. In the meta-analysis by Roumeau et al. (2022) [128], rhNGF achieved complete corneal healing in 75% of patients, comparable to outcomes with autologous serum (92%), amniotic membrane transplantation (AMT, 86%), and corneal neurotization (99%). In contrast, non-specific treatments, such as lubricants, were associated with a markedly lower healing rate of 23%. Surgical and non-surgical treatments demonstrated similar rates of complete healing overall. Notably, only rhNGF and AMT were associated with significant improvements in visual acuity. Moreover, rhNGF demonstrated a relatively short mean time to epithelial healing (24.2 days) compared to 27.6 days for autologous serum, 16.4 days for AMT, and 117 days for neurotization. Vera-Duarte et al. (2024) [129] also emphasized that rhNGF is distinct among therapies for neurotrophic keratopathy due to its ability to restore corneal epithelial integrity while promoting reinnervation, thereby addressing both the epithelial and neural components of the disease, unlike conventional treatments that primarily provide trophic support (e.g., autologous serum, amniotic membrane) or mechanical protection (e.g., artificial tears, bandage contact lenses) without targeting the underlying sensory nerve deficit.

In the case of autologous serum, which can mimic some effects of recombinant NGF therapy through its content of neurotrophic and epithelial-supporting factors such as NGF, epidermal growth factor (EGF), transforming growth factor-beta (TGF-β), insulin-like growth factor-1 (IGF-1), fibronectin, and vitamin A [130,131,132,133], its therapeutic benefits are likely multifactorial. Moreover, the concentration of NGF in autologous serum is several orders of magnitude lower than that achieved with pharmacologic formulations like cenegermin [130,131,132], which are specifically designed to reach effective levels for epithelial healing and corneal nerve regeneration. This difference may account for the greater efficacy of rhNGF in restoring visual acuity, as described above.

### 5.3. Immune Keratitis

Lambiase et al. (2000) examined the use of murine NGF in patients with autoimmune corneal ulcers and corneal melting unresponsive to immunosuppressive therapy [13]. In the study, four patients with severe corneal melting secondary to immune-related peripheral corneal ulcers received one drop of murine NGF solution (10 µg in 50 µL, equivalent to 200 µg/mL) every two hours (from 6 AM to 12 PM) for two days, followed by six times daily until the ulcer healed. Once complete healing was achieved, the dose was reduced to 5 µg in 50 µL (equivalent to 100 µg/mL), administered four times daily for two weeks. All patients demonstrated complete corneal healing within eight weeks of treatment.

### 5.4. Herpetic Keratitis

Murine NGF has been proposed as a potential therapy to treat and prevent the recurrence of herpetic keratitis. In a rabbit model, Lambiase et al. (2008) [15] demonstrated that topical administration of murine NGF significantly improved both clinical and laboratory parameters compared to the balanced salt solution (BSS)-treated control group. Notably, the therapeutic effects of NGF were comparable to those of acyclovir, with no significant differences observed between the two treatment groups. In contrast, treatment with neutralizing anti-NGF antibodies worsened disease severity, resulting in fatal HSV encephalitis in two animals. Animals in the study were divided into four groups and treated topically with either NGF, acyclovir, BSS, or neutralizing anti-NGF antibodies. Disease severity was graded using a keratitis scoring system: 0.0 to 0.5 indicated normal or non-specific superficial lesions; 0.6 to 0.9 indicated punctate ulcerations; 1.0 to 1.9 indicated dendritic ulcerations; 2.0 to 2.9 indicated geographic ulcerations or trophic erosions involving less than 50 percent of the cornea; and 3.0 to 3.9 indicated involvement of more than 50 percent of the cornea [14]. A correlation between NGF deficiency and HSV reactivation was also demonstrated in rabbits with latent HSV infection [134]. Although the underlying mechanism remains unclear, the authors suggested that NGF’s immunomodulatory effects, such as promoting T and B lymphocyte proliferation and regulating antibody production [135], may contribute to its ability to suppress HSV replication. Supporting its therapeutic potential, a case report [16] describing a 68-year-old HIV-positive male with an acyclovir-resistant herpetic corneal ulcer reported successful treatment with NGF eye drops. NGF was administered at a concentration of 200 μg/mL (10 μg NGF dissolved in 50 μL of 0.9% saline), instilled into the inferior conjunctival fornix every two hours until complete epithelial healing, which was achieved after 23 days. Following ulcer resolution, the drops were continued at the same concentration four times daily for an additional 15 days. No recurrence was observed during one year of follow-up.

Although the molecular mechanisms underlying NGF’s protective effects in herpetic keratitis are not yet fully understood, emerging evidence suggests that its therapeutic benefits arise from its immunomodulatory, anti-inflammatory, and neurotrophic properties rather than direct antiviral activity. In addition to the above-mentioned study by Lambiase et al. (2008) [15], recent in vitro studies suggest NGF has the capacity to inhibit Toll-like receptor 3 (TLR3)-mediated inflammatory cascades in human corneal epithelial cells by suppressing NF-κB activation and reducing reactive oxygen species (ROS) production, thereby attenuating the expression of pro-inflammatory cytokines such as IL-6, IL-8, IFN-β, and RANTES [136]. These findings hint at NGF’s ability to mitigate virus-induced immunopathology by dampening excessive inflammation through the modulation of innate immune signaling pathways, specifically TLR signaling, which is particularly relevant to HSV pathology. Additional mechanistic insights from diabetic keratopathy models highlight NGF’s ability to attenuate hyperglycemia-induced oxidative stress, inflammation, and apoptosis. In vitro and in vivo studies by Park et al. (2016) [137] demonstrated that NGF markedly reduced ROS generation, NF-κB activation, and the expression of IL-1β and TNF-α in corneal epithelial cells cultured under high-glucose conditions. NGF also suppressed the expression of cleaved caspase-3 and BAX, which are key markers of apoptosis, and significantly reversed corneal epithelial damage and inflammatory responses in streptozotocin-induced diabetic rats. These data reinforce the notion that NGF exerts broad cytoprotective effects and suggest possible mechanisms through which it mitigates viral diseases via suppression of inflammatory and apoptotic signaling. Lastly, given that NGF is both produced by and acts upon various immune cell types and is upregulated systemically and locally during inflammation, infection, and stress, it may act as a coactivator of immune responses and contribute to the recruitment, activation, and survival of immune cells, as suggested here [138,139]. Based on this, it is plausible that exogenously administered NGF could similarly modulate immune activity and enhance host responses during immunologic insults.

### 5.5. Dry Eye Disease

NGF and its receptors are expressed in the lacrimal gland, and NGF has been detected in human tear film [19]. Topical NGF administration has also been shown to stimulate tear secretion in patients with DED [18,25]. Chang et al. (2008) [17] further proposed that NGF may reduce corneal epithelial cell apoptosis and mitigate damage caused by chronic hyperosmolar stress, a hallmark of DED. As previously discussed, NGF may exert cytoprotective effects on the ocular surface through mechanisms relevant to dry eye disease. In vitro, NGF has been shown to reduce TLR3-mediated inflammation and oxidative stress, potentially via NF-κB inhibition and decreased ROS production, leading to lower expression of IL-6, IL-8, and IFN-β [136]. In diabetic keratopathy models, NGF also reduced apoptotic markers and improved epithelial integrity [137]. While not studied directly in DED, these pathways may contribute to epithelial preservation under desiccating stress.

Clinical studies involving human participants [17,140] have also demonstrated a positive correlation between tear NGF levels and dry eye severity. Using enzyme-linked immunoassays, researchers quantified NGF concentrations in tear samples from affected individuals and found correlations with conjunctival hyperemia, fluorescein staining, and OSDI scores greater than 20, thereby supporting the potential utility of NGF as a biomarker for disease severity in DED. A Phase II, prospective, open-label, multiple-dose clinical trial [141] evaluated the safety and efficacy of rhNGF eye drops in 40 patients with DED. This single-center study tested two concentrations of rhNGF eye drop solution (4 μg/mL and 20 μg/mL), administered twice daily in both eyes for 28 days. The outcomes assessed included treatment-emergent AEs; the frequency and severity of DED symptoms, evaluated using the Symptoms Assessment in Dry Eye (SANDE) scale; ocular surface staining assessed with lissamine green using the National Eye Institute scale; tear production measured by Schirmer test type I (without anesthesia); and tear function assessed by tear film break-up time and tear film osmolarity. Both concentrations of rhNGF induced significant improvement in the signs and symptoms of DED; however, improvement in tear film break-up time was observed only in the 20 μg/mL group. These findings suggest that higher concentrations of rhNGF significantly enhance tear production and function.

Interestingly, another Phase II multicenter randomized double-masked vehicle-controlled dose-ranging trial [142] evaluated the safety and efficacy of rhNGF (cenegermin) eye drops at a concentration of 20 µg/mL in 261 patients with moderate to severe DED, including Sjögren’s DED. Patients were randomized to receive cenegermin eye drops at a dose of 20 µg/mL either two or three times daily, or vehicle alone, for four weeks, followed by a 12-week follow-up period. Although the primary endpoint of change in Schirmer I score at week 4 was not met, a significantly higher proportion of patients treated with cenegermin achieved clinically meaningful tear production (Schirmer I greater than 10 mm in 5 min) compared to vehicle. Notably, only the group receiving cenegermin three times daily showed sustained and statistically significant improvements in patient-reported symptoms, as measured by the SANDE and (Impact of Dry Eye on Everyday Life) IDEEL questionnaires, throughout the follow-up period. Cenegermin was generally well tolerated, with mild and transient eye pain being the most commonly reported AE. These findings suggest that administering cenegermin 20 µg/mL three times daily may provide greater and longer lasting symptomatic relief in patients with moderate to severe DED.

### 5.6. Corneal Transplantation

In a preclinical study, Gong et al. (2007) [21] demonstrated that NGF gene therapy may enhance corneal graft survival by modulating immune responses. Using a high-rejection rat model, Lewis rats received corneal grafts from inbred female Dark Agouti donors, which are fully mismatched at major histocompatibility complex (MHC) class I and II loci, resulting in uniform graft rejection. A single local administration of adenovirus-mediated NGF gene transfer one day prior to transplantation significantly prolonged graft survival, preserved endothelial cell density, and suppressed the expression of key pro-inflammatory cytokines, including TNF-α, IFN-γ, IL-12p40, and IL-4, compared to untreated controls. Interestingly, NGF was shown to reduce IL-6 and IL-8 secretion in human corneal epithelial cells in vitro, possibly by modulating NF-κB signaling pathways [136]. While this effect has not been investigated in corneal transplantation models, IL-6 and IL-8 are known to be upregulated in the tear film and aqueous humor following procedures such as endothelial keratoplasty and Descemet’s membrane endothelial keratoplasty, where they contribute to postoperative inflammation and increased risk of graft rejection [143,144,145].

The use of rhNGF in NK following penetrating keratoplasty (PKP) was reported in a case involving a 24-year-old woman who had previously undergone acoustic neuroma surgery [20]. The patient presented with stage 3 NK, which necessitated multilayer amniotic membrane transplantation (AMT) and two PKPs due to recurrence. After the second PKP, 20 μg/mL rhNGF eye drops were administered six times daily for eight weeks, resulting in complete corneal epithelial healing and a stable ocular surface maintained over 12 months of follow-up. Pan et al. (2018) [146] investigated the association between tear NGF levels and corneal subepithelial nerve regeneration following keratoplasty. In this retrospective study, tear samples from 30 eyes of 28 patients undergoing primary keratoplasty were analyzed. NGF concentrations were reduced on postoperative day 1 compared to preoperative levels but showed a progressive increase on days 7, 30, and 90. By day 90, peripheral nerve buds were detected in 80% of the grafts, and their presence significantly correlated with elevated tear NGF levels, suggesting that postoperative upregulation of NGF may contribute to subepithelial nerve regeneration after keratoplasty. Finally, a case study by Gouvea et al. (2021) [147] reported the clinical outcomes of a 75-year-old male with lattice dystrophy and a history of HSV keratitis who developed NK in the right eye two years after undergoing PKP. Following multiple recurrences and failure of NK-specific treatments, including dehydrated amniotic membrane, therapeutic contact lens, and lateral tarsorrhaphy, the patient was started on 20 μg/mL rhNGF eye drops administered six times daily. By week 8, the corneal epithelium had completely healed, and visual acuity (VA) improved from 20/100 to 20/50. The ocular surface remained stable during a six-month follow-up.

### 5.7. Cataract Surgery

A randomized controlled trial investigated the efficacy of topical murine NGF versus 0.2% hyaluronic acid in promoting corneal wound healing after cataract surgery [22]. The primary objective was to assess whether murine NGF accelerates postoperative epithelial recovery. Patients in the NGF group received 1 drop of 200 μg/mL murine NGF solution (10 μg in 50 μL of 0.9% saline) instilled into the inferior conjunctival fornix every two hours between 6 AM and 12 PM for two weeks, followed by four times daily administration for an additional week. The control group received 0.2% hyaluronic acid on the same schedule. By day 21 postoperatively, anterior segment optical coherence tomography (OCT) revealed complete stromal healing in the NGF-treated group, with no residual injury detectable. In contrast, the control group exhibited persistent incision lines extending from the corneal surface to Descemet’s membrane. These findings suggest that topical murine NGF significantly accelerates corneal wound healing following cataract surgery.

NGF may support corneal epithelial repair after cataract surgery by activating PI3K/AKT and MEK/ERK pathways, which are key regulators of epithelial cell survival, proliferation, and migration. As described earlier, NGF stimulation in corneal epithelial cells leads to phosphorylation of AKT and ERK, facilitating cell cycle progression and promoting epithelial regeneration [105]. These findings are consistent with evidence linking PI3K/AKT signaling to corneal wound healing [107] and with studies demonstrating that NGF enhances epithelial cell migration and proliferation [36].

### 5.8. Refractive Surgery

A trial involving rhesus monkeys evaluated nerve regeneration, as well as NGF mRNA and protein expression, following laser in situ keratomileusis (LASIK) to correct −8.00 diopters of myopia [25]. NGF mRNA expression increased 5.4-fold on day 3, remained elevated at 2-fold above baseline on day 7, and normalized by three months after surgery. NGF protein levels decreased on days 3 and 7 after LASIK but returned to control levels by one month postoperatively. These early changes in NGF mRNA and protein levels correlated with the density of the corneal nerve plexuses. The authors proposed that these alterations may be associated with the initiation of nerve regeneration and the eventual recovery of corneal nerve plexuses. In comparative studies, tear NGF levels are highest following photorefractive keratectomy (PRK), followed by LASIK and small incision lenticule extraction (SMILE) [23,24,148]. Moreover, a positive correlation has been observed between tear NGF levels and the degree of myopia corrected with SMILE [24].

Topical administration of murine NGF following refractive surgery was evaluated by Joo et al. (2004) [149]. In their prospective, double-masked study in rabbits, murine NGF (200 mg in 1 mL of BSS) was applied directly to the exposed stromal bed immediately after photoablation and then to the corneal surface four times daily for three days. Murine NGF treatment was associated with a faster recovery of corneal sensitivity after LASIK compared to the control group. Similarly, Gong et al. (2021) investigated the effects of topical NGF on dry eye following LASIK [150]. In their study, rabbits that underwent LASIK were randomly assigned to one of three treatment groups: 200 μg/mL murine NGF, 0.2% hyaluronate, or normal saline. The authors reported accelerated recovery of subbasal and superficial stromal nerve densities, improved corneal sensitivity, and increased tear film break-up time in the murine NGF group at one and three months postoperatively.

A prospective, nonrandomized, comparative clinical trial by Lee et al. (2005) evaluated tear NGF levels following LASIK and PRK [148]. The study reported an increase in tear NGF levels after both refractive procedures, with a greater elevation observed in the PRK group. Early postoperative NGF levels in tears were associated with reduced postoperative corneal sensation, tear film break-up time, and Schirmer test values. The authors observed lower tear film break-up times at 1, 3, and 6 months in the LASIK group, whereas the PRK group exhibited higher Schirmer test values and improved corneal sensation at 3 and 6 months. The authors hypothesized that the differences observed were due to the higher postoperative NGF levels detected in the PRK group.

Two cases of NK following LASIK treated with rhNGF were reported by Habibi et al. (2021) [151], with both procedures having occurred 20 years prior to presentation. After 8 weeks of treatment, there was a marked improvement in VA, corneal sensitivity, and fluorescein corneal staining. Nonetheless, signs and symptoms recurred in both cases: at 3 months after therapy in the first and 1 month in the second. These findings suggest that a longer duration of treatment may be beneficial for patients with mild chronic manifestations of neurotrophic disease.

### 5.9. Adverse Events

AEs associated with murine NGF and rhNGF have been reported; however, all were transient [16,22,53,103,120,141,152]. Local AEs include eye pain (affecting 0–63.5%), foreign body sensation (4.3–20%), blurred vision (10.8–16.7%), photophobia (1.9–16.7%), increased lacrimation (0–15%), ocular pruritus (7.7%), eyelid pain (0–10.7%), eye discharge (0–5%), ocular hyperemia (0–4.3%), corneal deposits (0–4.3%), anterior chamber inflammation (4.3%), eye inflammation (4.3%), hyphema (4.3%), keratitis (4.3%), eye paresthesia (4.3%), posterior capsule opacification (4.3%), and corneal neovascularization (0–1.9%). Systemic AEs described in the literature include headache (1.9–25%), rhinitis (0–10%), nasopharyngitis (0–5%), back and neck pain (0–5%), tinnitus (0–5%), and flatulence (0–5%). To date, no serious AEs have been reported with the topical use of NGF on the ocular surface [10,11,141,142,152,153]. Following topical administration, a small proportion of NGF is absorbed by the conjunctiva, peri-orbital tissue, and cornea, with distribution mainly confined to the anterior segment [103]. rhNGF is eliminated through tears and the nasolacrimal duct, with minimal systemic absorption [52]. Notably, murine NGF and rhNGF do not induce the production of circulating NGF antibodies [103,120,152]. The most commonly reported AEs are related to topical application and may, in some cases, reflect corneal reinnervation and sensitivity [103]. A notable limitation in the current body of experimental research is the absence of data on the use of rhNGF in pregnant women [52].

A randomized, double-masked, vehicle-controlled trial [152] evaluated ocular and systemic AEs associated with rhNGF in healthy volunteers. Outcomes measured included blood chemistry, urinalysis, vital signs, electrocardiograms (ECGs), serum NGF antibody levels, ocular and systemic AEs, VA, tear function, intraocular pressure, fundus examination, and ocular symptoms. A few mild and transient ocular AEs related to rhNGF administration were reported, including a transient burning sensation, photophobia, and conjunctival hyperemia. These findings are consistent with those reported by Lambiase et al. (2007) [120] in NK patients treated with murine NGF. It is also noteworthy that a retrospective study in pediatric patients with NK found that 25% of those with pre-existing mild corneal neovascularization exhibited increased conjunctival injection and progression of corneal stromal neovascularization during rhNGF treatment [53]. These findings are consistent with those of Matsuyama et al. (2017) [154], who administered pellets containing murine NGF into 1–2 mm corneal pockets to assess the distribution and characteristics of perivascular nerves in neovascularized tissue using a mouse corneal micropocket model. Immunohistochemistry analysis revealed that NGF promotes the innervation of perivascular nerves, potentially regulating blood flow within neovessels and accelerating the maturation of pre-existing corneal blood vessels.

**Table 1:** Studies evaluating nerve growth factor as a treatment for ocular surface diseases.

Authors	Study Design	Ocular Surface Disease	No. of Patients (Eyes)	Type of NGF	Treatment Scheme	Outcomes	Follow-Up	Results
Lambiase et al. (1998) [68]	Case series	Neurotrophic keratopathy	12 (14)	Murine NGF	1 drop (200 μg/mL) every 2 h (6–12 AM) for 2 days, then 6 times daily until ulcer healed. 1 drop (100 μg/mL) 4 times daily for 2 weeks post-healing.	Corneal healing.	3 months	All patients had complete resolution of the corneal ulcer after 10 days to 6 weeks of treatment.
Bonini et al. (2000) [66]	Prospective, noncomparative, interventional case series	Neurotrophic keratopathy	43 (45)	Murine NGF	1 drop (200 μg/mL) every 2 h (6–12 AM) for 2 days, then 6 times daily until healed. 1 drop (100 μg/mL) 4 times daily for 2 weeks post-healing.	Size and depth of the ulcer or the epithelial defect, corneal sensitivity, best corrected VA, side effects, and relapse of the disease.	15.8 +/− 11.5 months	Complete resolution of the persistent epithelial defect after 12 days to 6 weeks of treatment in all patients. AE: Hyperemia and ocular and periocular pain.
Tan et al. (2006) [127]	Case report *	Neurotrophic keratopathy	1 (1)	Murine NGF	1 drop (200 μg/mL) every 2 h (6 AM–12 AM) for 2 days, then 6 times daily until healing. 1 drop (100 μg/mL) 6 times daily until a cumulative dose of 3 mg was reached.	Corneal healing.	4 months	Corneal healing at 8 weeks.
Bonini et al. (2018) [11,12]	Randomized controlled trial	Neurotrophic keratopathy	Phase I: 18 (18); Phase II: 156 (156)	rhNGF	1 drop (10 μg/mL or 20 μg/mL) 6 times daily for 8 weeks.	Corneal healing, AEs.	56 weeks	At week 8, 74.5% of patients receiving rhNGF 10 μg/mL had less than 0.5 mm of lesion staining compared to 74.0% of those receiving rhNGF 20 μg/mL. During follow-up, 96% of patients remained recurrence-free. AEs were mostly local, mild, and transient.
Pedrotti et al. (2019) [124]	Case report *	Neurotrophic keratopathy	1	rhNGF	1 drop (20 μg/mL) 6 times daily for 8 weeks.	Corneal healing.	21 weeks	Corneal healing at 3 weeks.
Pflugfelder et al. (2020) [10]	Multicenter randomized controlled trial	Neurotrophic keratopathy	48	rhNGF	1 drop (20 μg/mL or vehicle) 6 times daily for 8 weeks.	Corneal healing and sensitivity, changes in VA.	24 weeks	At week 8, 65.2% of patients in the 20 μg/mL rhNGF group achieved 0 mm of lesion staining with no residual staining compared to 16.7% in the control group.
Ahuja et al. (2020) [8]	Case report	Neurotrophic keratopathy	1	rhNGF	1 drop (20 μg/mL) 6 times a day for 8 weeks.	Corneal healing.	-	Corneal healing at 8 weeks.
Pocobelli et al. (2020) [20]	Case report	Neurotrophic keratopathy on penetrating keratoplasty	1	rhNGF	1 drop (20 μg/mL) 6 times a day for 8 weeks.	Corneal healing.	12 months	Complete corneal healing was achieved after 5 weeks. NK recurred at week 9. After a second cycle of treatment, no recurrence was observed during follow-up.
Fausto et al. (2020) [125]	Case report *	Neurotrophic keratopathy	1	rhNGF	1 drop (20 μg/mL) 6 times a day for 8 weeks.	Corneal healing.	6 months	Corneal healing was achieved at 8 weeks, with a reduction in corneal opacity and neovascularization.
Mandarà et al. (2022) [7]	Case report	Neurotrophic keratopathy	1	rhNGF	1 drop (20 μg/mL) 6 times a day for 8 weeks.	Corneal healing.	12 months	Corneal healing at 8 weeks.
Hatcher et al. (2021) [53]	Retrospective case series *	Neurotrophic keratopathy	8 (9)	rhNGF	1 drop (20 μg/mL) 6 times a day for 8 weeks.	Corneal healing, sensation and scarring, VA, and AEs.	2–13 months	63% experienced clinical improvement with no recurrence in 10 months. AEs: ocular pain, difficulty sleeping, continued corneal thinning, and corneal neovascularization.
Papadopoulos et al. (2021) [123]	Case report *	Neurotrophic keratopathy	1	rhNGF	1 drop (20 μg/mL) 6 times a day for 8 weeks.	Corneal healing.	8 months	Corneal healing was achieved at 8 weeks, with no recurrence during follow-up.
Habibi et al. (2021) [151]	Case series	Neurotrophic keratopathy following LASIK	2 (4)	rhNGF	1 drop (20 μg/mL) 6 times a day for 8 weeks.	Corneal healing, VA, and recurrence.	5 months	Corneal healing and VA improvement at 8 weeks. Recurrence at 1 and 3 months after treatment.
Cheung et al. (2022) [121]	Retrospective chart review	Neurotrophic keratopathy	16 (18)	rhNGF	8 weeks course, drop frequency not specified.	Corneal healing and sensitivity.	3–20 months	Corneal sensation increased from 7% to 79% of eyes. Among patients with a persistent epithelial defect, 67% experienced complete resolution.
Bruscolini et al. (2022) [9]	Retrospective chart review	Neurotrophic keratopathy	18	rhNGF	1 drop (20 μg/mL) 6 times daily for 8 weeks.	Corneal healing and sensitivity, VA, and recurrence.	48 months	Corneal healing was observed at 8 weeks, with improvements in corneal sensitivity, VA, and tear production during follow-up. Three cases recurred within 12 months and one case within 36 months.
Hamrah et al. (2024) [153]	Phase IV multicenter, prospective, open-label clinical trial	Neurotrophic Keratopathy (Stage 1)	37	rhNGF	1 drop (20 μg/mL) 6 times daily for 8 weeks.	Corneal epithelial healing (fluorescein staining). Secondary: corneal sensitivity, BCDVA, QoL measures (IDEEL, EQ-5D-5L), Schirmer, TFBUT.	5.5 months	At week 8, 84.8% showed corneal epithelial healing, with 95.2% remaining healed at week 32. Corneal sensitivity improved in 91.2% at week 8 and 82.1% at week 32. Mean BCDVA improved by −0.10 logMAR at week 8. Eye pain (37.8%) was the most common AE, typically mild or moderate.
Lambiase et al. (2000) [13]	Case series	Immune corneal ulcer	4 (5)	Murine NGF	1 drop (200 μg/mL) every 2 h (6 AM–12 PM) for 2 days, then 6 times daily until ulcer healed; 1 drop (100 μg/mL) 4 times daily for 2 weeks post-healing.	Corneal healing.	3–12 months	Corneal healing within 8 weeks of treatment.
Cellini et al. (2006) [22]	Randomized controlled trial	Cataract surgery	30 (30)	Murine NGF	NGF group: 1 drop (200 μg/mL) every 2 h (6 AM–12 PM) for 2 weeks, then 4 times daily for 1 week. Hyaluronic acid group: 1 drop (0.2%) every 2 h (6 AM–12 PM) for 2 weeks, then 4 times daily for 1 week.	Corneal thickness at the site of the surgical wound, the endothelial cell count, and the incision line in the stroma (via OCT).	21 days	No significant difference in endothelial cell count. The stromal incision was not visible at day 21 in the NGF group. Corneal thickness at day 21: NGF, 645.2 μm; HA, 704 μm.
Cellini et al. (2007) [16]	Case report	Herpetic keratitis	1	Murine NGF	1 drop (200 μg/mL, 10 μg/50 μL) every 2 h until ulcer healed (23 days), then 4 times daily for 15 additional days.	Corneal healing.	12 months	Corneal healing at 23 days.
Ferrari et al. (2014) [152]	Randomized controlled trial	Healthy volunteers	74	rhNGF	Single dose: 1 drop (0.0175 μg, 0.175 μg, or 0.7 μg rhNGF). Single-day dosing: 1 drop (2.1 μg, 6.3 μg, or 18.9 μg total/day) 3 times daily for 1 day. Multiple-day dosing: 1 drop (10.5 μg, 31.5 μg, or 94.5 μg total) 3 times daily for 5 days.	Blood chemistry, urinalyses, vital signs, ECGs, serum NGF antibodies, ocular and systemic AEs, visual acuity, tear function, intraocular pressure, fundus oculi, and ocular symptoms.	10 or 30 days	Treatment did not result in a significant increase in circulating NGF levels, and no antidrug antibodies were detected in serum. There was no detectable clinical evidence of systemic AEs. Ocular AEs were transient and mild in intensity.
Sacchetti et al. (2020) [141]	Phase IIa, prospective, open-label, multiple-dose, clinical trial	Dry eye disease	39 (78)	rhNGF	1 drop (4 μg/mL or 20 μg/mL) twice daily for 28 days.	AE, change in DED symptoms, staining and tear function.	8 weeks	The severity of DED symptoms and ocular surface damage showed significant improvement in both groups, while tear function improved only in the 20 µg/mL group.
Wirta et al. (2024) [142]	Phase II, randomized, double-masked, vehicle-controlled, dose-ranging trial	Dry eye disease (including Sjögren’s DED)	261 (522)	rhNGF	1 drop (20 µg/mL) two or three times daily for 28 days.	Schirmer I score, SANDE, IDEEL, TFBUT, ocular staining, AE.	12 weeks	Although the primary endpoint (Schirmer I score) was not met, significantly more patients in both rhNGF groups achieved Schirmer >10 mm. Only the three-times-daily group showed sustained symptom improvement. Cenegermin was well tolerated.

* Studies including pediatric patients.

## 6. Challenges of Nerve Growth Factor as an Ocular Surface Therapy

NGF is a potential therapeutic agent for various ocular surface diseases, particularly those involving epithelial regeneration (e.g., NK), inflammation (e.g., allergic conjunctivitis and immune keratitis), infection (e.g., HSV keratitis), and post-surgical corneal wound healing. NGF exerts downstream effects on neurons, fibroblasts, endothelial and smooth muscle cells, mast cells, eosinophils, and lymphocytes through multiple biochemical pathways. Its ability to modulate this diverse group of cells, each playing a critical role in both physiological and pathological states of the ocular surface, may explain the broad therapeutic applicability of NGF across a range of ocular surface conditions.

The clinical application of NGF in NK is the most extensively studied, with supporting evidence from both randomized controlled trials and basic science research. However, certain reported AEs warrant further investigation to clarify the relationship between topical NGF therapy and their occurrence. Notably, one patient in the 20 μg/mL group of the REPARO trial developed corneal neovascularization [11]. This observation aligns with findings from Hatcher et al. (2021) [53], who reported similar outcomes in a retrospective study involving pediatric patients. The effect of murine NGF and rhNGF on corneal neovascularization remains unclear and requires further evaluation through studies with extended follow-up. Ongoing research into the pathophysiological mechanisms of NGF on the ocular surface may help clarify the potential contribution of NGF to corneal neovascularization in selected cases.

Although NGF is recognized for its neuroprotective properties and its ability to support epithelial healing, its role in ocular surface disease appears to be context dependent. Elevated concentrations of NGF have been reported in the tear film of patients with contact-lens-associated dry eye, Sjögren’s syndrome, ocular cicatricial pemphigoid, keratoconjunctivitis sicca, and dry eye following refractive surgery [19,99,131,140,148,155], suggesting that NGF is not consistently deficient across disease states. In such chronic inflammatory conditions, increased NGF levels may contribute to disease persistence by sustaining immune cell activation and inflammation. The occurrence of corneal neovascularization during rhNGF therapy [11,53] also raises concerns about a potential proangiogenic effect in susceptible patients. Finally, while activation of the high-affinity TrkA^NGFR^ receptor is generally associated with beneficial outcomes such as nerve regeneration and epithelial repair through PI3K/Akt and MEK/ERK signaling, unopposed or excessive engagement of the low-affinity p75^NTR^ receptor may initiate proapoptotic and pro-inflammatory cascades via the JNK and NF-κB pathways [75,90,94,97,105,106,107,156]. Thus, although NGF upregulation may initially aid tissue repair, prolonged or dysregulated signaling may drive nerve dysfunction, chronic inflammation, and tissue remodeling.

The cost-effectiveness and availability of rhNGF are important factors that may pose challenges to its widespread use as a therapy for ocular surface diseases, particularly in publicly funded healthcare systems. The Liverpool Reviews and Implementation Group at the University of Liverpool was commissioned by the National Institute for Health and Care Excellence (NICE) to serve as the evidence review group in evaluating the clinical and cost-effectiveness of cenegermin (rhNGF) for NK. The group found no evidence of long-term benefit, and as a result, it was not possible to establish a reliable estimate of cost-effectiveness [157]. Finally, the administration regimen is complex, requiring eye drops to be instilled up to six times per day over an 8-week treatment period [10,11].

There are several limitations in the current literature on NGF use on the ocular surface. Extrapolating the results to the general population is challenging due to heterogeneous study samples and designs, as well as the limited number of clinical studies focused on specific ocular surface diseases included in this review. Randomized controlled trials evaluating NGF in the context of NK or DED are scarce, and much of the existing data is derived from case reports and small case series. As a result, a substantial portion of the literature consists of studies with low levels of evidence [158]. Inadequate long-term follow-up is another limitation of existing trials. From a clinical perspective, future studies would benefit from incorporating survival analyses (e.g., Kaplan–Meier estimates) to assess recurrence rates in NK patients at 6 to 12 months post-treatment. Future studies, particularly randomized controlled trials, are warranted for a range of conditions in which evidence for NGF use remains limited, such as DED, refractive surgery, and allergic conjunctivitis, among others. Moreover, the role of NGF in these conditions is not yet fully understood. Drug-induced and iatrogenic insults, including chronic use of preservative-containing eye drops, topical anesthetic abuse, and ocular surgery (e.g., cataract, refractive, and corneal transplant surgeries), can also lead to impairments of corneal innervation and epithelial integrity, contributing to DED, NK and various other diseases of the ocular surface [20,66,148,151,159,160]. Given NGF’s roles in nerve regeneration, epithelial repair, and stimulation of tear secretion, its therapeutic efficacy in these underexplored contexts warrants further investigation. Although the efficacy of topical NGF has been notable in several studies on NK, further research is needed to guide clinicians in identifying optimal clinical scenarios and appropriate treatment regimens, especially considering that cost remains a barrier to routine use. A more comprehensive understanding of the underlying pathophysiological mechanisms of NGF may facilitate its development as a therapeutic agent for a wider range of ocular surface diseases.

## 7. Conclusions

NGF has been studied across a range of contexts, from basic science research to nonrandomized and randomized clinical trials. In primary human conjunctival epithelial cells and cell line cultures, NGF has been shown to increase goblet cell numbers, enhance mucin precursor production and storage, and support neural innervation and maintenance [49]. Clinically, topical administration of NGF has demonstrated positive outcomes in various ocular surface diseases, with NK being the most extensively studied. Notably, rhNGF is the first FDA-approved topical biologic therapy in the field of ophthalmology, specifically indicated for the treatment of NK. Altogether, NGF is a molecule with notable therapeutic effects on the ocular surface. It regulates tear production and supports the survival and proliferation of epithelial cells, fibroblasts, immune cells, endothelial cells, and corneal sensory nerves. Both experimental and clinical evidence support its role as a potential treatment and biomarker for degenerative and immune-mediated diseases, as well as for post-surgical wound healing of the ocular surface. Addressing the current limitations of NGF-based therapy through well-designed, large-scale, multicenter randomized controlled trials with long-term follow-up across diverse clinical settings may expand its approved indications. Moreover, such efforts may establish rhNGF as a first-line therapy rather than a rescue option for cases unresponsive to conventional treatment.

## Data Availability

No new data were created or analyzed in this study.
